# AI-based tumor and organ volumetry predicts early progression after Y-90 radioembolization in liver-dominant metastatic disease

**DOI:** 10.1007/s00259-026-08051-6

**Published:** 2026-07-04

**Authors:** Daniel Martinez Heinemann, Jannis Bodden, Lukas Endroes, Felianty Teresa, Manuel Nickel, Sebastian Lange, Markus Mergen, Daniel Spitzl, Roland Schmid, Marc Martignoni, Jan Kirschke, Matthias Eiber, Rickmer Braren, Lisa Steinhelfer

**Affiliations:** 1 School of Medicine and Health, Department of Diagnostic and Interventional Neuroradiology, Technical University of Munich, TUM University Hospital, Munich, Germany; 2 Department of Diagnostic and Interventional Radiology and Nuclear Medicine, University Medical Center Hamburg-Eppendorf, Hamburg, Germany; 3 School of Medicine and Health, Department of Radiology, Technical University of Munich, TUM University Hospital, Munich, Germany; 4 Chair for AI in Healthcare and Medicine, Technical University of Munich (TUM), TUM University Hospital, Munich, Germany; 5 TUM School of Medicine and Health, Clinical Department of Internal Medicine II, TUM University Hospital, Munich, Germany; 6 Department of Surgery, Klinikum Rechts der Isar, School of Medicine and Health, Technical University of Munich, Munich, Germany; 7 School of Medicine and Health, Department of Nuclear Medicine, Technical University of Munich, TUM University Hospital Munich, Munich, Germany; 8 Bavarian Cancer Research Center (BZKF), Erlangen, Germany; 9 Partner Site Munich, A Partnership between DKFZ and School of Medicine, German Cancer Consortium (DKTK), Technical University of Munich, Munich, Germany

**Keywords:** Liver metastases, TARE, Yttrium-90, AI-based volumetry, Tumor volume, Imaging biomarker, Progression-free survival

## Abstract

**Purpose:**

In patients with liver-dominant metastatic disease undergoing yttrium-90 (⁹⁰Y) transarterial radioembolization (TARE), response assessment remains challenging, particularly in multifocal disease where manual RECIST-based measurements may not adequately reflect overall hepatic tumor burden. In addition, the prognostic relevance of volumetric changes in tumor, liver, and spleen after TARE remains unclear. This retrospective study evaluated automated CT-based whole-liver tumor volumetry as a quantitative imaging biomarker for response assessment and progression risk stratification following TARE.

**Methods:**

We retrospectively analyzed imaging and clinical data from 57 adults with liver-dominant metastatic disease (colorectal, neuroendocrine, uveal melanoma, and breast cancer) treated with yttrium-90 (⁹⁰Y) resin TARE between January 2012 and December 2022. Inclusion criteria were age ≥ 18 years, availability of baseline contrast-enhanced CT imaging, and clinical follow-up. Patients undergoing liver resection, transplantation, or additional locoregional therapies during follow-up were excluded.

**Results:**

Baseline tumor volume was a strong independent predictor of progression-free survival (PFS), demonstrating excellent discriminatory performance (ROC-AUC 0.91; 95% CI 0.82–1.00). A threshold of 270 mL most accurately identified early progression (< 12 months), with a sensitivity of 0.84 and specificity of 0.92. In contrast, lesion count, embolization strategy, and maximum tumor diameter were not significantly associated with PFS.

**Conclusions:**

Automated baseline tumor volumetry demonstrated promising prognostic performance compared with conventional clinical and procedural parameters in patients with liver-dominant metastases treated with ⁹⁰Y TARE. Automated quantification of intrahepatic tumor burden may serve as a useful and readily accessible imaging biomarker for early risk stratification, although external validation is required before clinical implementation.

**Supplementary Information:**

The online version contains supplementary material available at https://doi.org/10.1007/s00259-026-08051-6.

## Introduction

Liver metastases are a major contributor to cancer-related morbidity and mortality, as progressive intrahepatic tumor burden can compromise hepatic function and limit systemic treatment options [1]. In several solid tumors, liver metastases are common and frequently represent a major determinant of prognosis, making durable intrahepatic disease control an important therapeutic objective, even in palliative settings [2, 3]. Transarterial radioembolization (TARE) with yttrium-90 (⁹⁰Y) microspheres is a form of intra-arterial brachytherapy that delivers high-dose beta radiation to liver tumors via the hepatic arterial circulation, enabling selective irradiation of tumor-bearing territories while relatively sparing uninvolved liver parenchyma [4, 5]. Evidence from randomized trials, meta-analyses, and systematic reviews supports ⁹⁰Y radioembolization as a liver-directed option across secondary hepatic malignancies [6–10]. Although TARE is generally well tolerated, clinically significant toxicity can occur from excessive irradiation of non-tumorous liver parenchyma [11, 12].

Accurate and reproducible quantification of tumor burden and treatment response remains challenging in patients with multifocal metastatic liver disease. Conventional response assessment in solid tumors primarily relies on unidimensional measurements according to RECIST 1.1, which may inadequately reflect the total intrahepatic tumor burden, particularly in the presence of multifocal disease [13]. In patients undergoing radioembolization for liver metastases, pretreatment tumor burden has consistently been reported as an independent prognostic factor, including in colorectal cancer liver metastases and hepatic metastases from uveal melanoma [8, 14]. Moreover, splenic volume has been proposed as a quantitative imaging biomarker associated with progression-free survival (PFS) after TARE, particularly in hepatocellular carcinoma, further supporting the broader role of volumetric imaging parameters in risk stratification and outcome prediction [15]. However, manual volumetric assessment of liver, spleen, and tumor lesions remains time-consuming, labor-intensive, and prone to interobserver variability, particularly in complex tumor regions, limiting its integration into routine clinical workflows. Recent advances in deep learning have enabled automated volumetric assessment with integration into clinical imaging workflows [16]. In particular, TotalSegmentator, based on nnU-Net architectures, allows robust automated segmentation, thereby facilitating scalable volumetric biomarker analyses in routine imaging datasets [17, 18]. Automated volumetric quantification integrated into routine imaging workflows enables the longitudinal assessment of quantitative morphologic imaging biomarkers, including organ volumes and tumor burden. In this context, the present study evaluates AI-derived tumor, hepatic, and splenic volumetry as potential imaging biomarkers for predicting PFS in patients with liver-dominant metastatic disease undergoing TARE.

## Materials and methods

### Patients

All patients underwent standardized pretreatment evaluation, including clinical assessment, laboratory testing, and contrast-enhanced CT imaging. Inclusion criteria for this retrospective analysis were age ≥ 18 years; liver-dominant metastatic disease from prespecified primary tumor entities (colorectal cancer, neuroendocrine tumors, uveal melanoma, or breast cancer); and treatment with TARE between January 2012 and December 2022. Liver-dominant disease was defined as predominant hepatic tumor involvement relative to extrahepatic sites. Eligible patients had available baseline and 3-month follow-up CT, corresponding laboratory data, and a minimum clinical follow-up of 3 months. Patients undergoing liver resection, transplantation, or additional locoregional therapies during follow-up were excluded. Treatment indication was established by multidisciplinary tumor board consensus. All patients underwent preprocedural angiography and technetium-99 m–labeled macroaggregated albumin (⁹⁹ᵐTc-MAA) simulation to evaluate hepatic arterial anatomy and hepatopulmonary or extrahepatic shunting. Prophylactic embolization was performed when indicated to prevent non-target radiation (gastroduodenal artery, *n* = 1; right gastric artery, *n* = 1). TARE was conducted using yttrium-90 (⁹⁰Y) resin microspheres (SIR-Spheres; Sirtex Medical, Sydney, Australia), with activity calculated according to the body surface area method as previously described [19, 20]. Embolization strategy (lobar, segmental, subsegmental), administered activity, and lung shunt fraction were recorded. Ethical approval for this retrospective analysis was obtained from the local institutional review board (reference 87/18S). Patient selection is illustrated in Supplementary Figure S1.

### Automated image segmentation and analysis

**Fig. 1:**
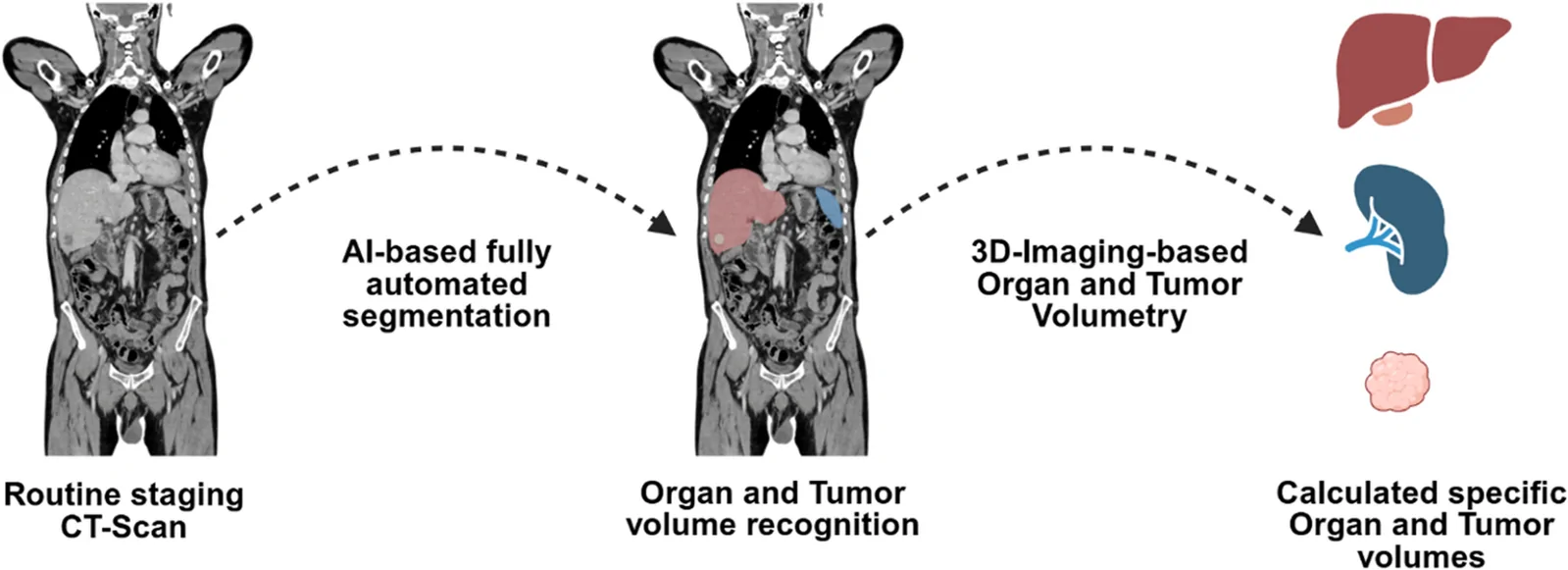
AI-based CT segmentation and volumetric quantification. Routine staging CT scans were processed to perform automated 3D segmentation of the liver, spleen, and intrahepatic tumor burden. All segmentations underwent expert review, with manual correction when necessary. The resulting segmentations were used to derive quantitative measurements of organ and tumor volumes

Baseline and 3-month follow-up contrast-enhanced CT scans were used for volumetric assessment. Automated organ segmentation was performed using the publicly available deep learning model TotalSegmentator [17] on axial CT images (contrast-enhanced and/or non-contrast) with slice thickness ≤ 5 mm when available. Total liver and spleen volumes were extracted from the segmentation masks. Intrahepatic tumor burden was quantified using automated lesion segmentation, based on an nnU-Net–based open-source contribution to TotalSegmentator [21], followed by expert review and manual correction when necessary, resulting in a cumulative tumor volume per scan (Fig. 1). All segmentations were reviewed by two board-certified radiologists (L.S., 6 years; R.B., 18 years of experience). Absolute volumes at baseline and 3 months, as well as absolute and relative changes, were calculated. Functional imaging–based dosimetric surrogates were derived from pretherapeutic ⁹⁹ᵐTc-MAA SPECT/CT. Tumor-to-normal liver ratio (TNr) was calculated according to established guideline-based methodology [4].

### Follow-up and endpoints

Follow-up imaging was performed according to institutional routine, with the first post-treatment CT planned at approximately 3 months after TARE. Subsequent imaging examinations were performed at comparable, although not standardized, intervals as clinically indicated during routine oncologic follow-up. Routine monitoring included alanine aminotransferase (ALT), aspartate aminotransferase (AST), γ-glutamyl transferase (GGT), albumin, bilirubin, hemoglobin (Hb), white blood cell (WBC) count, and platelet (PLT) count at baseline and approximately 3 months after TARE. Baseline and 3-month Child–Pugh score, MELD score, and ALBI grade were recorded, and relative changes from baseline to 3 months were calculated. The primary endpoint was PFS, defined as the interval between TARE and the first radiologically documented intrahepatic disease progression. For landmark analyses, patients were categorized as early progression (PFS < 12 months) versus late progression (PFS ≥ 12 months).

### Statistical analysis

All statistical analyses were performed in R (version 4.5.0). Continuous variables are reported as median with interquartile range (IQR), and categorical variables as counts with percentages. Between-group comparisons according to early progression status were performed using the Wilcoxon rank-sum test for continuous variables and Fisher’s exact test for categorical variables. PFS was analyzed using Cox proportional hazards regression. Given the number of candidate clinical, laboratory, and imaging variables, all prespecified predictors were initially evaluated in univariate Cox models. Variables meeting a liberal exploratory screening threshold of *p* < 0.10 were considered for inclusion in multivariable models to avoid excluding potentially relevant predictors. Due to the limited sample size and number of progression events, multivariable analyses were restricted to minimize overfitting. As the study was exploratory, no formal correction for multiple testing was applied. Multicollinearity among candidate predictors was assessed using variance inflation factors (VIF), and variables with VIF > 5 were excluded. The proportional hazards assumption was evaluated using Schoenfeld residual diagnostics, and hazard ratios (HRs) are reported with 95% confidence intervals. Baseline and 3-month tumor volumes reflect the same underlying construct measured at closely related time points and were therefore not included simultaneously in multivariable models to avoid multicollinearity. Accordingly, two prespecified models were evaluated: Model A including baseline tumor volume (primary analysis) and Model B including 3-month tumor volume (analysis reported in the Supplement). The tumor-to-liver ratio was assessed in univariate analyses but excluded from multivariable models to avoid collinearity, as it represents a derived metric based on the primary volumetric variables. Tumor volume was scaled per 100 mL to facilitate interpretation of HR estimates. Receiver operating characteristic (ROC) analysis was used to identify clinically interpretable thresholds for 12-month PFS (PFS ≥ 12 months vs. < 12 months). Optimal cutoffs were determined using the Youden index, and discrimination was summarized by the area under the curve (AUC) with 95% confidence intervals calculated using DeLong’s method. To assess the internal stability of the ROC-derived baseline tumor volume cutoff, 2,000 bootstrap resamples were generated. Within each resample, the Youden index-derived cutoff was recalculated and applied to the corresponding out-of-bag observations to estimate sensitivity and specificity; AUC was calculated for baseline tumor volume in the out-of-bag samples. Kaplan–Meier curves were generated for groups defined by ROC-derived cutoffs and compared using the log-rank test. A two-sided α level of 0.05 was considered statistically significant.

## Results

Baseline characteristics are summarized in Table 1. A total of 57 patients with liver metastases who underwent TARE with yttrium-90 (⁹⁰Y) resin microspheres (SIR-Spheres; Sirtex Medical, Sydney, Australia) between January 2012 and December 2022 were included in this retrospective analysis. Median age was 61 years (IQR 50–71), and 31 patients (54%) were male. Primary tumor entities were colorectal cancer (42.1%), neuroendocrine tumors (38.6%), uveal melanoma (10.5%), and breast cancer (8.8%); 79% had undergone prior resection of the primary tumor. All patients received a single TARE cycle. The median pre-treatment lung shunt fraction was 5.3% (IQR 3.9–6.6%). Extrahepatic metastases were present in a subset of patients, most frequently involving the lungs (26.3%), bone (21.1%), and lymph nodes (19.3%). Most patients had multifocal liver involvement (84.3%), while 15.7% presented with unifocal disease. Baseline liver function was largely preserved, with Child–Pugh class A in 96.5% and class B in 3.5% of patients. According to the ALBI score, 84.2% were classified as grade A and 15.8% as grade B. The median MELD score was 6 (IQR 6–8). Radioembolization was performed at the lobar level in 70.2% of cases, segmental in 24.5%, and subsegmental in 5.3%. The right hepatic lobe was treated in 89.5% of patients and the left lobe in 71.9%, with bilobar treatment performed in 14%. The median administered activity was 1.84 GBq (IQR 1.40–2.33).

**Table 1:** Baseline characteristics of patients included in the study (*n* = 57). Values are presented as median with the interquartile range presented in parenthesis. *For categorical features, absolute value counts are given with percentage in parenthesis. Abbreviations: ALBI = albumin–bilirubin grade; MELD = Model for End-Stage Liver Disease; PLT = platelets; WBC = white blood cell count; AST = aspartate aminotransferase; ALT = alanine aminotransferase; GGT = gamma-glutamyl transferase; PFS = progression-free survival

Clinical Characteristics		Value
Age [years]⁺	61 (50–71)	
Sex		
Male*	31 (54%)	
Female*	26 (46%)	
Primary tumor		
NET*	22 (38.6%)	
Colorectal*	24 (42.1%)	
Uveal melanoma*	6 (10.5%)	
Breast*	5 (8.8%)	
Primary tumor resection*	45 (79%)	
Metastatic sites		
Liver*	57 (100%)	
Lymphatic*	11 (19.3%)	
Bone*	12 (21.1%)	
Pulmonary*	15 (26.3%)	
Embolization strategy		
lobar*	40 (70.2%)	
segmental*	14 (24.5%)	
subsegmental*	3 (5.3%)	
Lesion pattern		
multifocal*	48 (84.3%)	
unifocal*	9 (15.7%)	
Baseline Child-Pugh Score		
A*	55 (96.5%)	
B*	2 (3.5%)	
C*	0 (0%)	
Baseline ALBI grade		
A*	48 (84.2%)	
B*	9 (15.8%)	
C*	0 (0%)	
Baseline MELD Score⁺	6 (6–8)	
Baseline Liver Volume [mL]⁺	1719 (1496–2083)	
Baseline Spleen Volume [mL]⁺	346 (290–393)	
Baseline Tumor Volume [mL]⁺	222 (134–325)	
Baseline Albumin Level [g/dL]⁺	4.30 (4.00–4.50)	
Baseline Bilirubin Level [mg/dL]⁺	0.40 (0.40–0.60)	
Baseline PLT [10^3^/µL]⁺	253 (185–298)	
Baseline WBC [10^3^/µL]⁺	7.22 (5.81–8.22)	
Baseline Hemoglobin [g/dL]⁺	12.60 (11.10–13.40)	
Baseline AST Level [U/L]⁺	43 (30–61)	
Baseline ALT Level [U/L]⁺	28 (21–53)	
Baseline GGT Level [U/L]⁺	112 (60–197)	
TARE Activity [GBq]⁺	1.84 (1.40–2.33)	

A total of 114 CT scans (57 baseline and 57 follow-up) were processed using TotalSegmentator. Automated segmentation of the liver, spleen, and intrahepatic tumor burden was successful in 109 scans (95.6%). Minor manual corrections were required in 5 cases (4.4%), including three instances of under-segmentation and two of over-segmentation, mainly related to misclassification of adjacent vascular structures such as the portal vein. At baseline, median liver volume was 1719 mL (IQR 1496–2083), median spleen volume 346 mL (IQR 290–393), and median intrahepatic tumor volume 222 mL (IQR 134–325). The median PFS was 13.0 months (IQR 10.0–18.0). The median imaging follow-up duration was 13 months (IQR 11–18), and the median interval between TARE and first follow-up CT was 94 days (IQR 82–109). Overall, 38 patients (66.7%) achieved a PFS of ≥ 12 months, whereas 19 patients (33.3%) experienced disease progression within 12 months.

### Survival analyses and prognostic modelling

During follow-up, all 57 patients experienced progression, and no observations were censored. The primary multivariable model included 57 patients and two covariates, corresponding to 28.5 progression events per covariate. Univariate Cox proportional hazards analyses across all tested clinical, laboratory, and imaging-derived features are provided in Table S1. Among the 53 features evaluated, five met the prespecified exploratory screening threshold of *p* < 0.10. Three variables were included in the multivariable analysis (Table 2). Tumor-to-liver ratio at baseline and at 3 months was excluded as a derived volumetric parameter to minimize collinearity, as outlined in the Methods section. Tumor volume demonstrated a strong association with PFS both at baseline and at 3 months: baseline tumor volume (per 100 mL) showed HR 2.11 (95% CI 1.58–2.82), *p* < 0.001, and 3-month tumor volume (per 100 mL) showed HR 2.16 (95% CI 1.61–2.91), *p* < 0.001. Liver volume at 3 months also met the screening threshold (*p* = 0.065). All other evaluated parameters, including laboratory markers, functional scores and imaging characteristics, did not show significant associations with PFS in this dataset.

Notably, the number and intrahepatic distribution of metastatic lesions, maximum tumor diameter at baseline and at three months (including percentage change), tumor volume change at three months, embolization strategy, and the tumor-to-normal liver uptake ratio (TNr) derived from 99mTc-MAA SPECT/CT were not significantly associated with PFS. To evaluate whether tumor volume remained predictive in a multivariable setting, we constructed Model A including baseline tumor volume and liver volume at three months (Table 2). Baseline tumor volume (per 100 mL) remained independently associated with PFS (HR 2.04, 95% CI 1.51–2.74; *p* < 0.001), whereas liver volume at 3 months was not significant (*p* = 0.326). A corresponding model including 3-month tumor volume (Model B) is shown in Table S2. Similarly, 3-month tumor volume (per 100 mL) remained independently associated with PFS (HR 2.10, 95% CI 1.54–2.86; *p* < 0.001), while liver volume at 3 months was not significant (*p* = 0.495). Baseline tumor volume was selected for presentation in the main manuscript, as it is available at the time of treatment planning and enables baseline risk stratification without reliance on follow-up imaging. Corresponding analyses using 3-month tumor volume, reflecting early post-treatment disease dynamics, are provided in the Supplement (Table S2; Figures S2–S4).

**Table 2:** Uni- and multivariable Cox proportional hazards (baseline tumor volume model). The multivariable model included 57 evaluable patients and 57 progression events. Cox regression results for baseline tumor volume (per 100 mL) and liver volume at 3 months (per 100 mL). Tumor and liver volumes were scaled per 100 mL to aid interpretability; estimates can be converted to per 1 mL by taking the 100th root of the HR. HR = hazard ratio; CI = confidence interval; *p* = *p*-value; PFS = progression-free survival. Bold *p*-values indicate statistical significance at *p* < 0.05

Feature	Univariate Cox Proportional Hazard			Multivariate Cox Proportional Hazard		
	HR	CI	*p*	HR	CI	*p*
Baseline Tumor Volume (per 100 mL)	2.11	1.58–2.82	**< 0.001**	2.04	1.51–2.74	**< 0.001**
Liver Volume at 3 months (per 100 mL)	1.07	1.00–1.15	0.065	1.04	0.97–1.11	0.326

### Tumor volume cutoffs for 12-month PFS (ROC analysis)

**Fig. 2:**
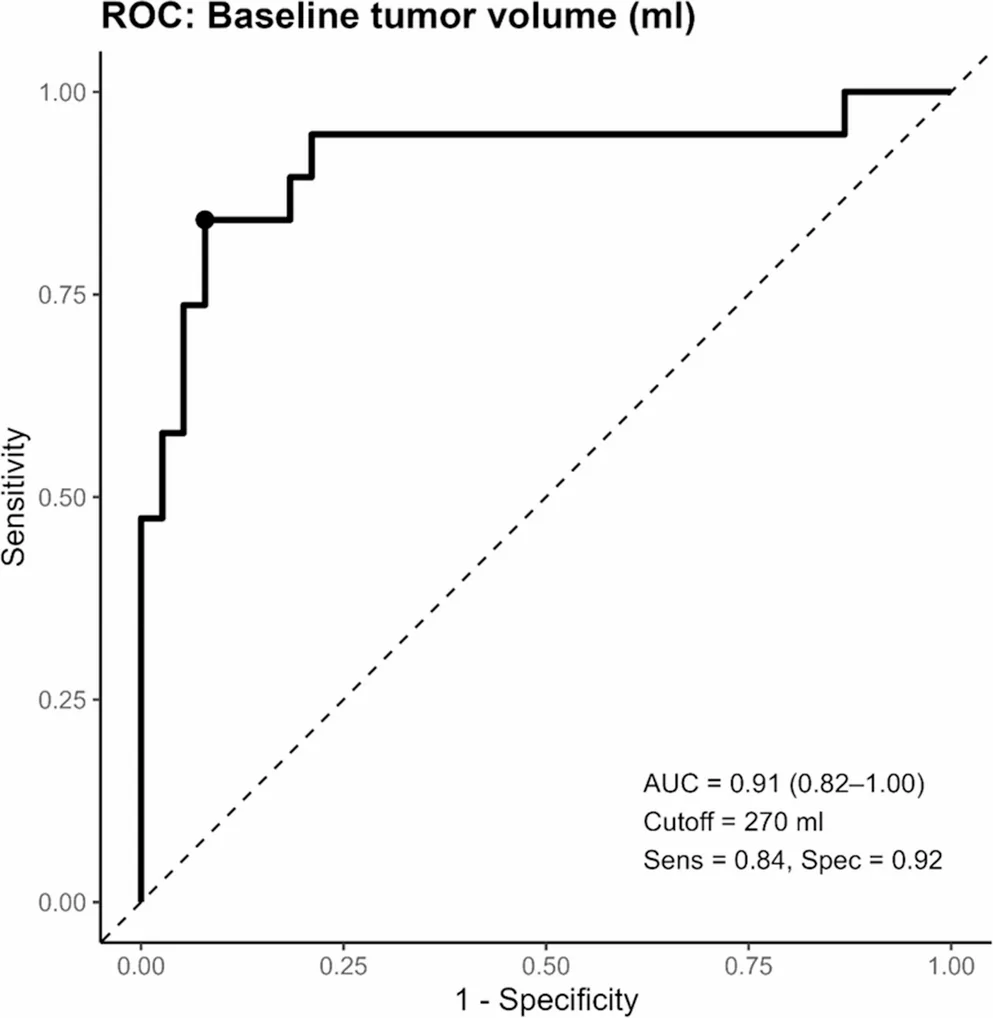
Baseline tumor volume: Receiver operating characteristic (ROC) analysis for prediction of 12-month PFS Receiver operating characteristic analysis was used to evaluate baseline intrahepatic tumor volume as a discriminator of early progression, defined as PFS < 12 months. The in-sample ROC-AUC was 0.91 (95% CI 0.82–1.00), with a Youden index-derived cutoff of 270 mL, sensitivity of 0.84, and specificity of 0.92. The cutoff should be interpreted as exploratory because it was derived within the same cohort

Given the clinical relevance of a 12-month PFS milestone, a commonly used landmark time point to summarize survival-type outcomes in oncology trials [22, 23], patients were stratified into PFS ≥ 12 months and PFS < 12 months to provide a pragmatic landmark for durable disease control after TARE. This threshold is consistent with prior radioembolization studies in metastatic liver disease [6, 24] and approximated the median time to progression in our cohort (13 months, IQR 10–18). Receiver operating characteristic (ROC) analyses were performed to determine tumor volume thresholds discriminating PFS ≥ 12 months from PFS < 12 months. For baseline tumor volume, the AUC was 0.91 (95% CI 0.82–1.00), with an optimal cutoff of 270 mL (sensitivity 0.84, specificity 0.92; Fig. 2). In 2,000 bootstrap resamples, the median re-derived baseline tumor volume cutoff was 266.5 mL (IQR 227.5–269.6 mL; 95% bootstrap percentile interval 226.1–318.9 mL). Across the corresponding out-of-bag evaluations, the median AUC was 0.93 (IQR 0.86–0.97), with a median sensitivity of 0.83 (IQR 0.75–1.00) and specificity of 0.88 (IQR 0.79–0.93). For 3-month tumor volume, the AUC was 0.89 (95% CI 0.79–1.00), with an optimal cutoff of 283 mL providing sensitivity 0.74 and specificity 0.95 (Figure S2).

### Kaplan–Meier analyses using ROC-derived cutoffs

**Fig. 3:**
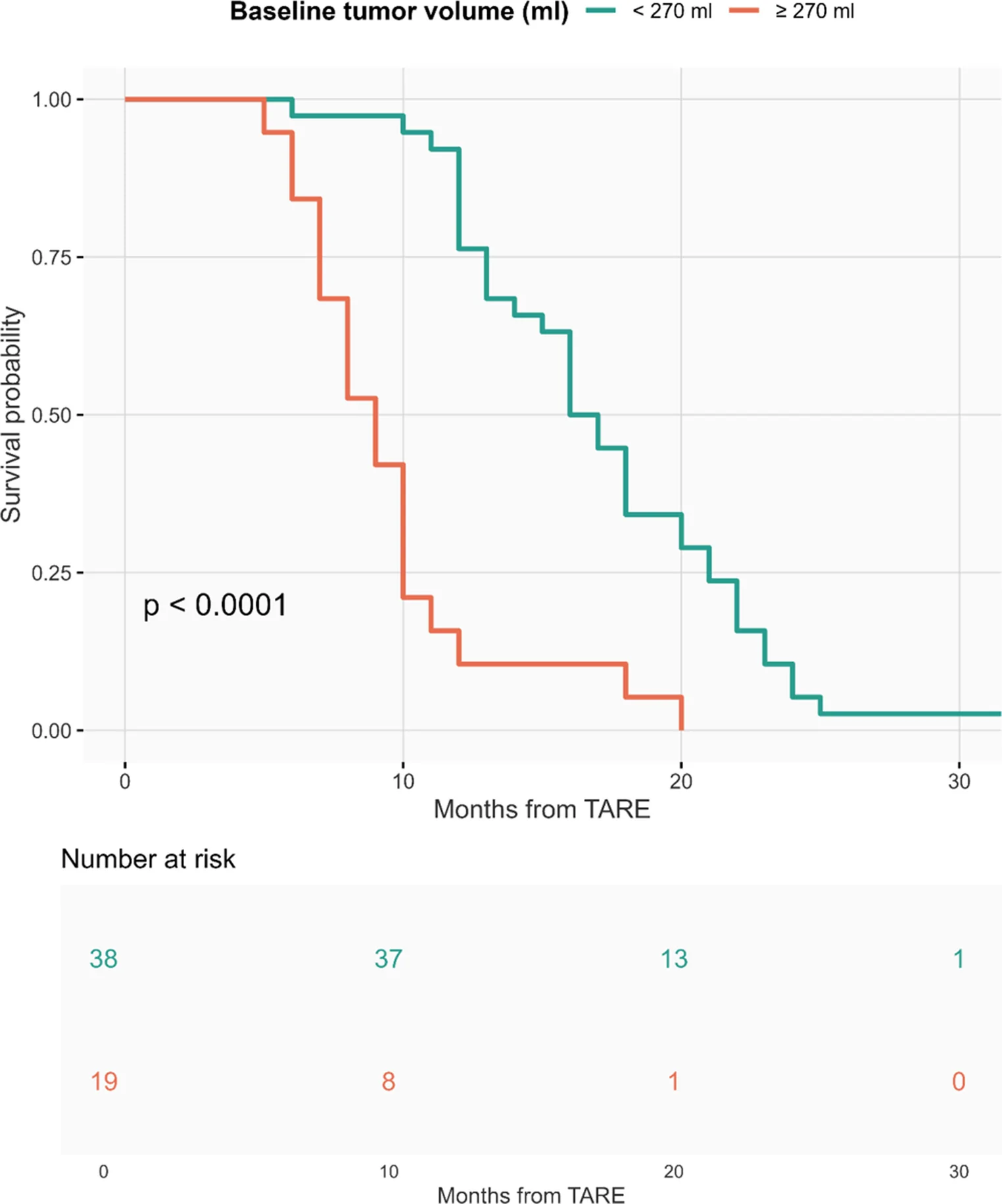
Kaplan–Meier analysis stratified by baseline tumor volume. Progression-free survival after TARE is shown for patients with baseline tumor volume < 270 mL (green) and ≥ 270 mL (orange)

Kaplan–Meier analyses demonstrated marked separation of PFS curves when stratifying patients by the ROC-derived thresholds. Median PFS was 17.0 months (95% CI 15.0–20.0) in patients with baseline tumor volume < 270 mL versus 8.0 months (95% CI 7.0–10.0) in those with baseline tumor volume ≥ 270 mL (log-rank *p* < 0.0001, Fig. 3).

### Distribution of tumor volumes by PFS group

**Fig. 4:**
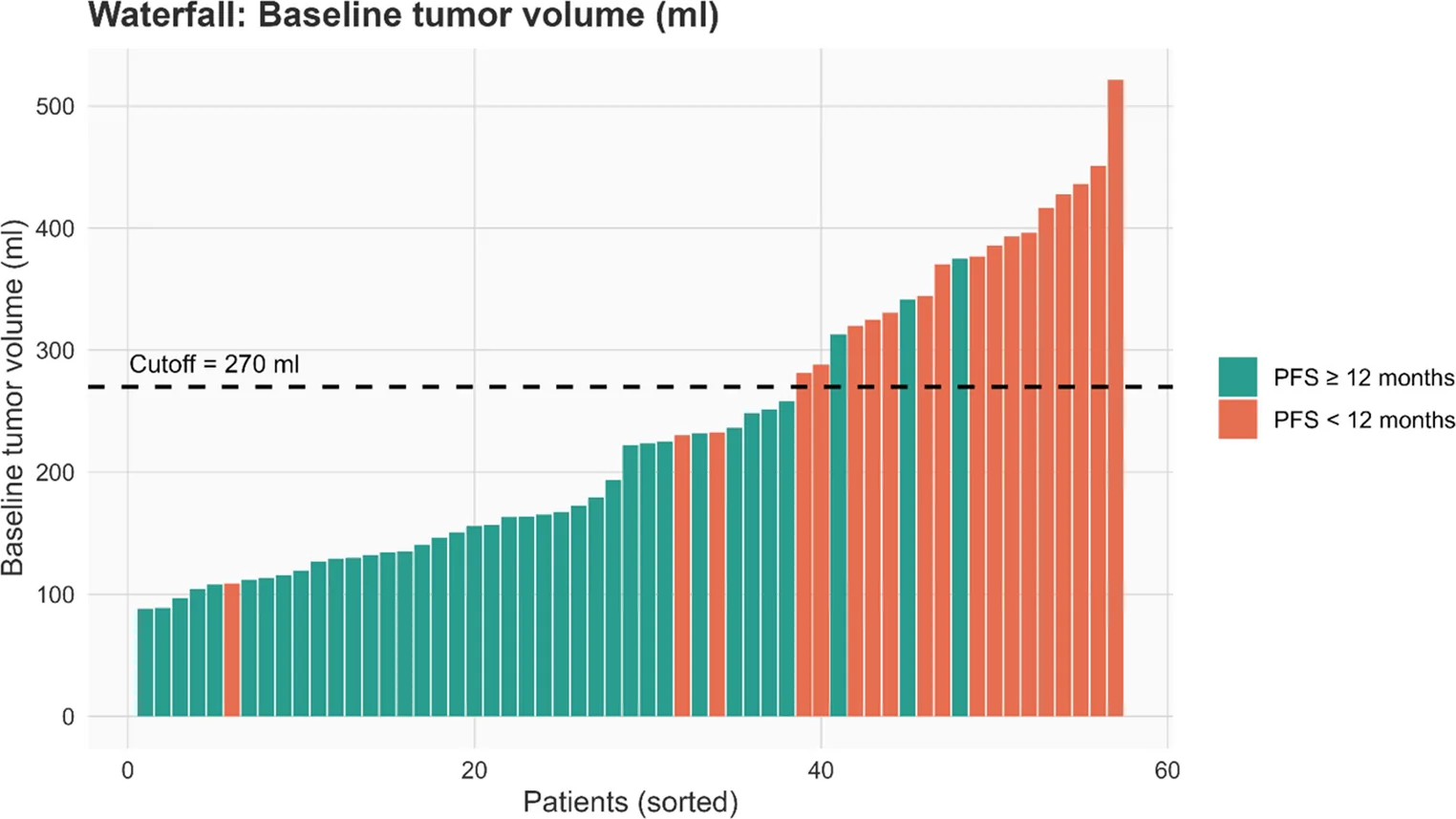
Waterfall plot of baseline tumor volume by PFS group Baseline tumor volume values are shown for all patients and sorted in ascending order; bar colors indicate PFS group (PFS ≥ 12 months vs. PFS < 12 months). The dashed line denotes the ROC-derived exploratory cutoff of 270 mL. Using this threshold, patients with early progression (< 12 months) were distinguished from those with sustained disease control (ROC-AUC 0.91, 95% CI 0.82–1.00; sensitivity 0.84; specificity 0.92)

Waterfall plots illustrate the distribution of baseline tumor volume across the cohort, with bars colored by PFS group (PFS ≥ 12 months vs. PFS < 12 months) and the ROC-derived cutoff shown as a dashed line. These plots demonstrate that patients experiencing early progression cluster at higher tumor volumes, consistent with the ROC and Kaplan–Meier results (Fig. 4).

## Discussion

In this retrospective single-center cohort of 57 patients with liver-dominant metastases treated with ⁹⁰Y resin radioembolization, we investigated whether automated CT-based volumetric parameters obtained at baseline and follow-up could serve as imaging biomarkers for predicting progression. In addition to imaging metrics, routinely used clinical and laboratory parameters, including AST, ALT, GGT, bilirubin, albumin, and established liver function indices such as ALBI grade, Child–Pugh score, and MELD score, were analyzed for their association with PFS. In univariate Cox regression analyses, tumor volume at baseline and at 3 months was strongly associated with PFS. Liver volume at 3 months also met the predefined threshold for inclusion in the multivariable model (*p* < 0.10). Among baseline variables, only tumor volume was predictive of PFS. In contrast, baseline liver and spleen volume, and clinical staging systems, including the Child-Pugh score, MELD score and ALBI grade, were not significantly associated with PFS.

Due to the absence of a widely accepted prognostic model for this patient population and the limited prognostic performance of established clinical parameters after TARE, we did not evaluate volumetric changes relative to a predefined reference model. Instead, all variables were first assessed in univariate analyses, and parameters meeting the prespecified exploratory screening threshold were considered for inclusion in multivariable Cox proportional hazards models. Baseline and 3-month tumor volumes were both strongly associated with PFS but highly correlated and were therefore evaluated in separate prespecified multivariable models. The tumor-to-liver ratio was analyzed only as a supportive univariate and sensitivity metric, as its inclusion alongside absolute volumes would introduce collinearity. In this multivariable analysis, baseline total tumor volume was identified as a strong independent predictor of PFS.

Stratification using a 12-month PFS threshold, a clinically relevant milestone time point frequently used to report survival outcomes in oncology trials [22, 23], identified a total tumor volume of 270 mL as the optimal cut-off to differentiate patients with early progression (< 12 months), from those achieving sustained disease control (PFS ≥ 12 months). This threshold demonstrated strong discriminatory performance, with a sensitivity of 0.84 and a specificity of 0.92, supporting the potential of total tumor volume as a non-invasive imaging biomarker for early outcome stratification prior to TARE. Bootstrap resampling yielded a median re-derived cutoff close to the original 270 mL threshold and preserved good discriminatory performance in out-of-bag evaluations.

The inclusion of multiple metastatic tumor entities was intended to evaluate total intrahepatic tumor burden as a common, clinically accessible imaging biomarker in a real-world TARE cohort. However, differences in tumor biology, vascularity, growth kinetics, and treatment history may modify the association between tumor volume and outcome. Accordingly, the identified cutoff should be regarded as exploratory and may not be directly transferable across individual primary tumor entities.

Our findings are consistent with previous radioembolization studies in metastatic disease, which have identified hepatic tumor burden as an important determinant of disease control and survival [14]. Accordingly, higher baseline tumor burden has repeatedly been associated with poorer outcomes following radioembolization [8, 25, 26]. Previous studies in metastatic colorectal cancer have identified higher pretreatment total liver tumor volume as a predictor of poorer response to ⁹⁰Y resin microsphere radioembolization [27]. Moreover, pretreatment FDG-PET-derived volumetric parameters, including metabolic tumor volume and total lesion glycolysis, have been associated with PFS and OS in patients undergoing ⁹⁰Y radioembolization for metastatic liver disease [28]. Interestingly, in contrast to previous reports, particularly in hepatocellular carcinoma (HCC) cohorts, where splenic volume and its post-therapeutic changes have been associated with PFS and overall survival [15, 29], neither baseline spleen volume nor longitudinal spleen volume changes were associated with PFS in our cohort. This difference may be explained by the absence of liver cirrhosis in our cohort. In patients with hepatocellular carcinoma (HCC), splenomegaly and post-therapeutic increases in splenic volume are closely associated with portal hypertension and impaired liver function. In this context, limited hepatic reserve increases the risk of treatment-related liver decompensation [30], which may explain why splenic volume has prognostic relevance in cirrhotic populations but not in our cohort. Likewise, the tumor-to-normal liver uptake ratio (TNr) derived from pretherapeutic ⁹⁹ᵐTc-MAA SPECT/CT was not associated with PFS. Although ⁹⁹ᵐTc-MAA is routinely used for treatment planning and predictive dosimetry as a surrogate for subsequent ⁹⁰Y microsphere distribution [31, 32], its accuracy is limited by differences in flow dynamics, catheter positioning, and particle characteristics between simulation and treatment [33–35]. These limitations, together with the biological heterogeneity of our cohort, may explain the limited prognostic value of TNr alone. Neither the number of metastases nor maximum tumor diameter were associated with PFS. This likely reflects limitations of conventional two-dimensional CT measurements in capturing true tumor burden. When tumor volume is inferred from linear measurements, a spherical geometry is assumed, with volume proportional to the cube of the diameter. Consequently, maximum diameter may underestimate differences in tumor burden, particularly in larger lesions. Moreover, as malignant tumors rarely exhibit spherical shapes, diameter-based estimates remain imprecise, and assessing only the largest lesion in patients with multiple metastases fails to capture cumulative tumor burden [13, 36]. Similarly, conventional liver function parameters - including ALT, AST, GGT, MELD and Child–Pugh scores, and ALBI grade - were not associated with PFS. This highlights limitations of established scoring systems such as the Child–Pugh score, which includes subjective components like ascites and encephalopathy that are prone to interobserver variability and influenced by non-hepatic factors [37]. The MELD score relies only on bilirubin, INR, and creatinine and may therefore insufficiently reflect hepatic function, particularly in patients with renal impairment unrelated to liver disease [38]. In addition, bilirubin and albumin levels may be influenced by factors beyond hepatic reserve (e.g., hemolysis for indirect bilirubin; systemic inflammation, malnutrition, or protein loss for albumin), which can limit specificity in heterogeneous metastatic cohorts [39, 40].

In this study, total tumor volume emerged as a strong independent predictor of PFS after TARE in patients with liver-dominant metastases. Three-dimensional volumetric assessment of intrahepatic tumor burden provided clinically meaningful prognostic information and may therefore serve as a valuable imaging biomarker for baseline risk stratification prior to TARE. Importantly, automated volumetry can be readily applied to routine clinical imaging datasets, supporting the feasibility of integrating volumetric tumor burden assessment into clinical workflows. However, prognostic imaging biomarkers may differ substantially between metastatic liver disease and primary liver tumors. This suggests that treatment planning and response assessment for TARE may require entity-specific imaging biomarkers, particularly in clinical scenarios where treatment sequencing - such as the transition from locoregional to systemic therapy - remains challenging.

Several limitations warrant consideration. First, the retrospective single-center design introduces potential selection and information bias, which may limit the generalizability of the findings. Formal validation of tumor segmentation accuracy against a manual reference standard and assessment of interobserver reproducibility were not performed and should be addressed in future studies. Second, the relatively small sample size restricts statistical power, particularly for subgroup analyses according to primary tumor type. Given the number of candidate variables assessed in this limited cohort, model instability and false-positive findings cannot be excluded. In the absence of adjustment for multiple testing, the results should be interpreted as exploratory. Furthermore, exploratory subgroup analyses by primary tumor entity were not performed because the individual subgroups, particularly uveal melanoma and breast cancer, were too small for reliable modelling. Third, the cohort comprised patients with heterogeneous tumor biology, disease stages, and systemic therapy histories across different tumor entities. Although this reflects real-world clinical practice, such heterogeneity may dilute or obscure entity-specific predictors. Therefore, the prognostic relevance of a given absolute tumor volume may differ across tumor entities. Although bootstrap resampling provided an internal assessment of the ROC-derived cutoff, the 270 mL threshold was derived from a small, single-center cohort and should not be regarded as a validated clinical decision boundary. Although the first post-treatment CT was planned at approximately 3 months, subsequent imaging intervals were not strictly standardized. Because progression could only be documented at the time of imaging assessment, this may have introduced assessment-time bias. Finally, our heterogeneous cohort limits interpretation of ⁹⁹ᵐTc-MAA SPECT/CT–derived TNr, as tracer distribution may differ across metastatic entities. Interpretation of TNr was further limited by the absence of personalized dosimetry, as absorbed dose metrics may provide complementary prognostic information after TARE. Future larger multicenter studies are needed to externally validate tumor volume thresholds, assess their incremental prognostic value beyond established clinical variables, and explore entity-specific models as well as clinically relevant endpoints, particularly outcomes after TARE in patients with predominant intrahepatic tumor burden.

## Conclusion

In conclusion, AI-based tumor volumetry is feasible in routine imaging datasets and may serve as a clinically useful biomarker for progression risk after ⁹⁰Y radioembolization in patients with liver-dominant metastatic disease. Baseline intrahepatic tumor volume was independently associated with PFS and showed strong discrimination between early and late progression, with a threshold of 270 mL. These findings support automated tumor burden quantification as a scalable tool for baseline risk stratification before TARE. At the same time, prognostic imaging biomarkers may differ between metastatic liver disease and primary liver tumors, suggesting the need for entity-specific approaches in TARE planning and response evaluation.

## Supplementary Information

Supplementary Material 1.

## Data Availability

The datasets generated during and/or analysed during the current study are available from the corresponding author on reasonable request.
