# Supplementary material for: AI-based tumor and organ volumetry predicts early progression after Y-90 radioembolization in liver-dominant metastatic disease
Source: Eur J Nucl Med Mol Imaging. 2026 Jul 4;53(12):6955–65. doi: 10.1007/s00259-026-08051-6 (PMC13633334; doi:10.1007/s00259-026-08051-6)
Supplement: Supplementary file 1 [file 259_2026_8051_MOESM1_ESM.pdf]

SUPPLEMENTAL MATERIAL

Feature	HR (95% CI)	p
Tumor Volume baseline (per 100 ml)	2.11 (1.58–2.82)	<0.001
Tumor Volume 3 months (per 100 ml)	2.16 (1.61–2.91)	<0.001
Δ-TV (3m - baseline)	1.00 (0.99–1.01)	0.895
%-TV 3 months	1.01 (0.99–1.03)	0.390
Max tumor diameter baseline [cm]	1.00 (0.97–1.02)	0.806
Max tumor diameter 3 months [cm]	1.00 (0.98–1.02)	0.755
%-Change max tumor diameter (3 months)	1.00 (1.00–1.00)	0.533
Tumor-to-normal liver ratio (TNr)	1.00 (0.87–1.16)	0.985
Tumor-to-liver ratio baseline	2.57 (1.60–4.15)	<0.001
Tumor-to-liver ratio 3 months	2.73 (1.69–4.42)	<0.001
Liver Volume baseline [ml]	1.00 (1.00–1.00)	0.104
Liver Volume 3 months (per 100 ml)	1.07 (1.00–1.15)	0.065
Δ-LV (3m - baseline)	1.00 (1.00–1.01)	0.347
%-LV 3 months	1.04 (0.96–1.12)	0.342
Spleen Volume baseline [ml]	1.00 (1.00–1.00)	0.541
Spleen Volume 3 months [ml]	1.00 (1.00–1.00)	0.724
Δ-SV (3m - baseline)	1.00 (1.00–1.00)	0.242
%-SV 3 months	1.00 (0.99–1.00)	0.310
Hemoglobin baseline [g/dL]	0.91 (0.78–1.06)	0.233
Hemoglobin 3 months [g/dL]	0.90 (0.77–1.06)	0.216
WBC baseline [10^3/µL]	0.93 (0.82–1.05)	0.244
WBC 3 months [10^3/µL]	0.97 (0.85–1.11)	0.639
Bilirubin baseline [mg/dL]	0.67 (0.23–1.96)	0.462
Bilirubin 3 months [mg/dL]	1.12 (0.53–2.34)	0.766
%-Change Bilirubin 3 months	1.00 (1.00–1.00)	0.517
Albumin baseline [g/dL]	0.96 (0.49–1.85)	0.893
Albumin 3 months [g/dL]	0.90 (0.42–1.91)	0.787

Feature	HR (95% CI)	p
%-Change Albumin 3 months	1.00 (0.98–1.02)	0.970
ALT baseline [U/L]	1.00 (0.99–1.01)	0.465
ALT 3 months [U/L]	1.00 (0.99–1.01)	0.900
AST baseline [U/L]	1.00 (0.99–1.01)	0.865
AST 3 months [U/L]	1.00 (1.00–1.01)	0.403
GGT baseline [U/L]	1.00 (1.00–1.00)	0.771
GGT 3 months [U/L]	1.00 (1.00–1.00)	0.362
PLT baseline [10^3/µL]	1.00 (1.00–1.00)	0.666
PLT 3 months [10^3/µL]	1.00 (1.00–1.00)	0.656
%-Change PLT 3 months	1.00 (0.99–1.02)	0.759
Ascites baseline	1.21 (0.55–2.69)	0.634
Ascites 3 months	1.58 (0.56–4.45)	0.391
Child-Pugh score baseline	1.84 (0.44–7.66)	0.405
Child-Pugh score 3 months	1.53 (0.47–5.01)	0.479
ALBI Score baseline	0.88 (0.40–1.94)	0.747
ALBI Score 3 months	1.14 (0.48–2.68)	0.764
ALBI Grade baseline	0.83 (0.40–1.70)	0.609
ALBI Grade 3 months	1.07 (0.51–2.28)	0.853
MELD Score baseline	1.03 (0.94–1.13)	0.471
Right liver lobe metastasis	0.94 (0.37–2.40)	0.905
Left liver lobe metastasis	0.79 (0.44–1.41)	0.424
Embolization strategy: segmental	0.98 (0.52–1.83)	0.945
Embolization strategy: subsegmental	0.38 (0.12–1.25)	0.112
Liver cirrhosis	0.39 (0.10–1.64)	0.200
Age [years]	0.99 (0.97–1.01)	0.208
Sex	1.29 (0.76–2.19)	0.341

Table S1. Univariate Cox proportional hazards (all tested features). Univariate Cox regression results for clinical, laboratory, and volumetric imaging features (N = 53). Tumor volume was scaled per 100 mL to aid interpretability; estimates can be converted to per 1 mL

by taking the 100th root of the HR. HR = hazard ratio; CI = confidence interval; p= P-value; PFS = progression-free survival.

	Univariate Cox Proportional Hazard			Multivariate Cox Proportional Hazard		
Feature	HR	CI	p	HR	CI	p
3-month Tumor Volume (per 100 ml)	2.16	1.61–2.91	**<0.001**	2.1	1.54–2.86	**<0.001**
Liver Volume 3 months (per 100 ml)	1.07	1.00–1.15	**0.065**	1.02	0.96–1.10	0.495

Table S2. Uni- and multivariable Cox proportional hazards (3-month tumor volume model). Cox regression results for 3-month tumor volume (per 100 mL) and liver volume at 3 months (per 100 mL). Tumor volume was scaled per 100 mL to aid interpretability; estimates can be converted to per 1 mL by taking the 100th root of the HR. HR = hazard ratio; CI = confidence interval; p = p-value; PFS = progression-free survival.

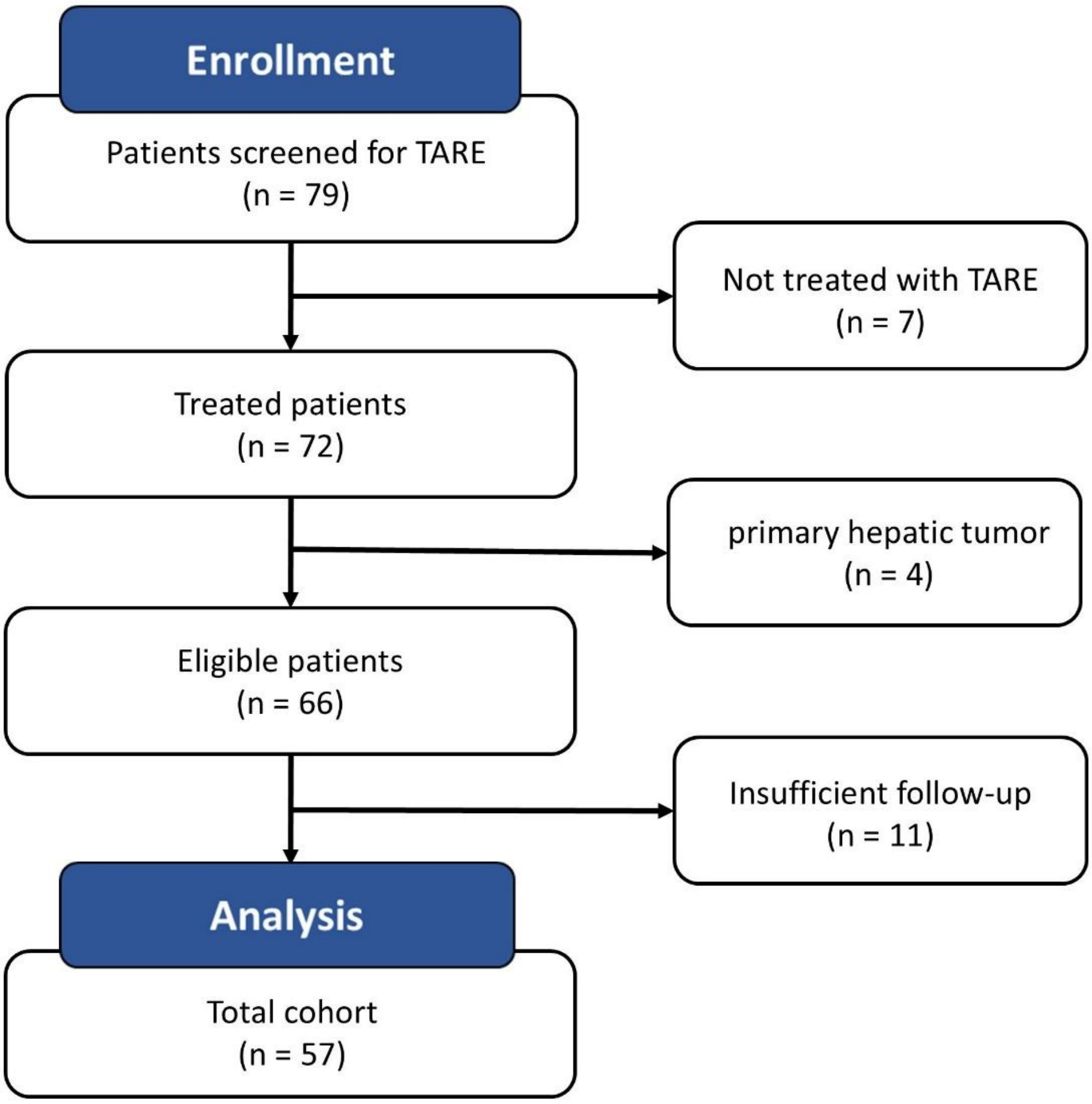

Figure S1. Patient inclusion flowchart. From 79 patients screened for transarterial radioembolization (TARE), 72 received TARE. Four patients with primary hepatic tumors were excluded, yielding 66 eligible patients. An additional 11 patients were excluded due to insufficient follow-up, resulting in a final study cohort of 57 patients included in the analysis.

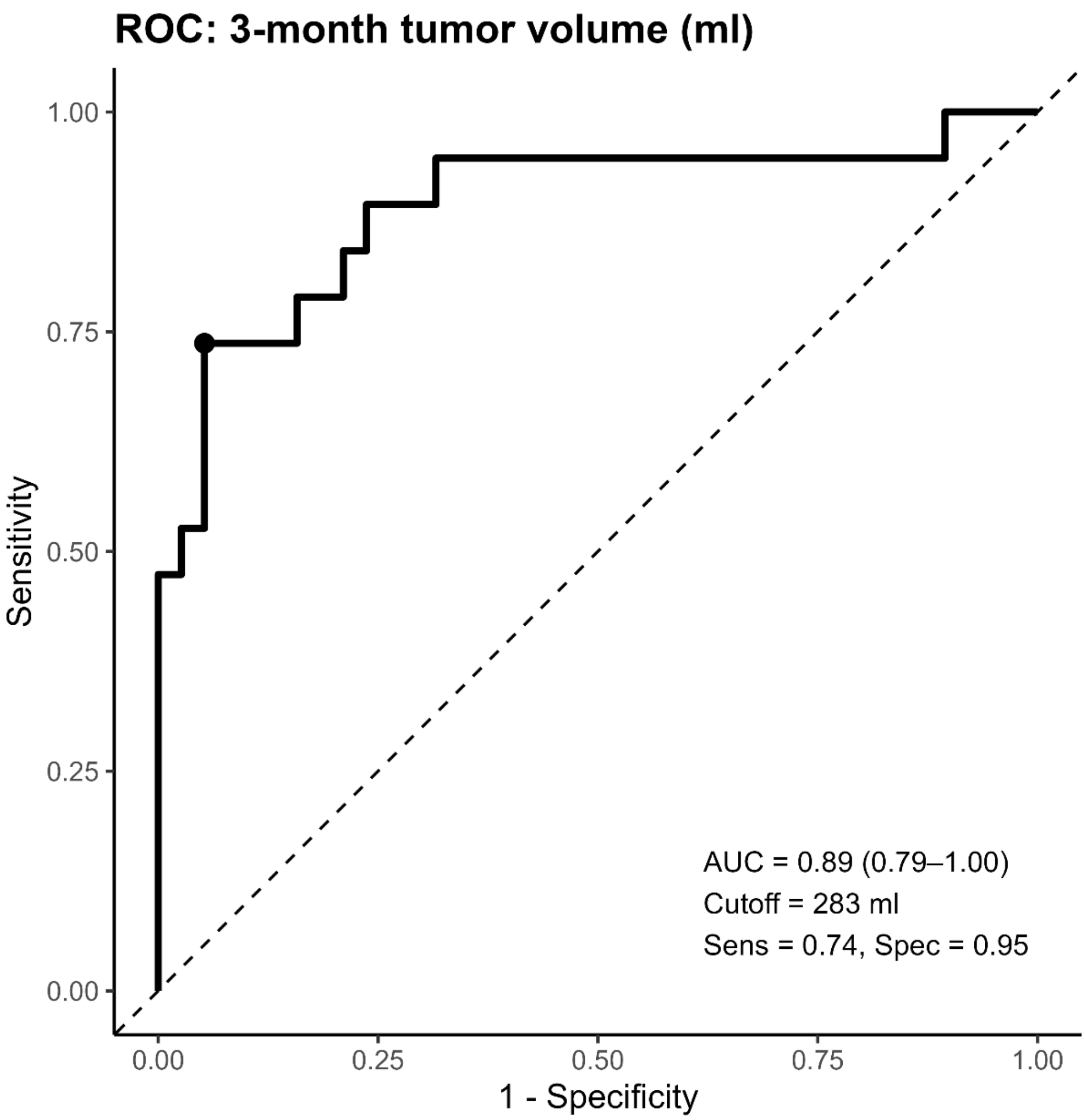

Figure S2. 3-month tumor volume: Receiver operating characteristic (ROC) analysis for prediction of 12-month PFS.
3-month tumor volume: AUC 0.89 (95% CI 0.79–1.00), cutoff 283 mL, sensitivity 0.74, specificity 0.95.

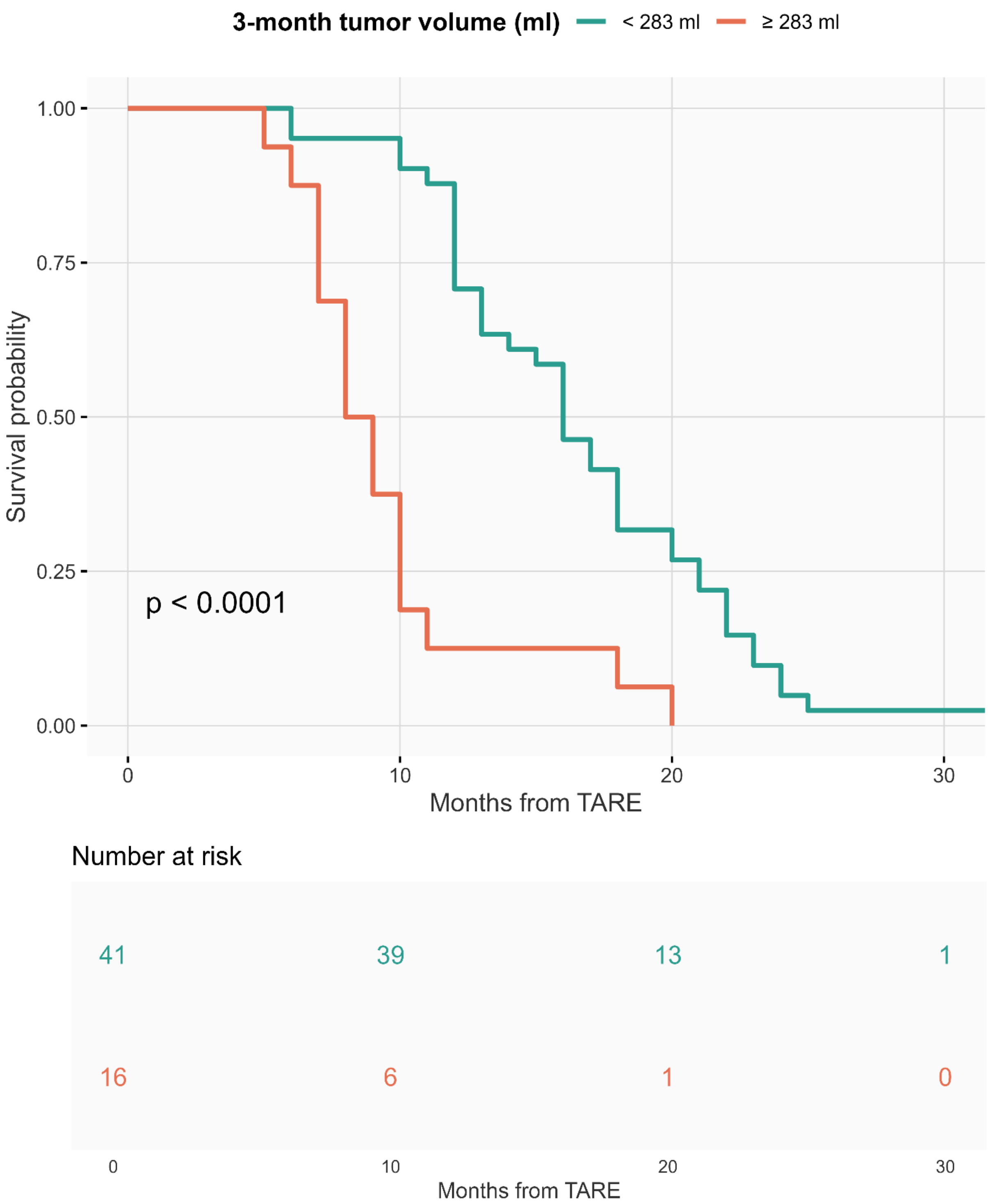

Figure S3. Kaplan–Meier analysis of progression-free survival stratified by tumor volume.

3-month tumor volume < 283 mL versus ≥ 283 mL. P values were calculated using the log-rank test.

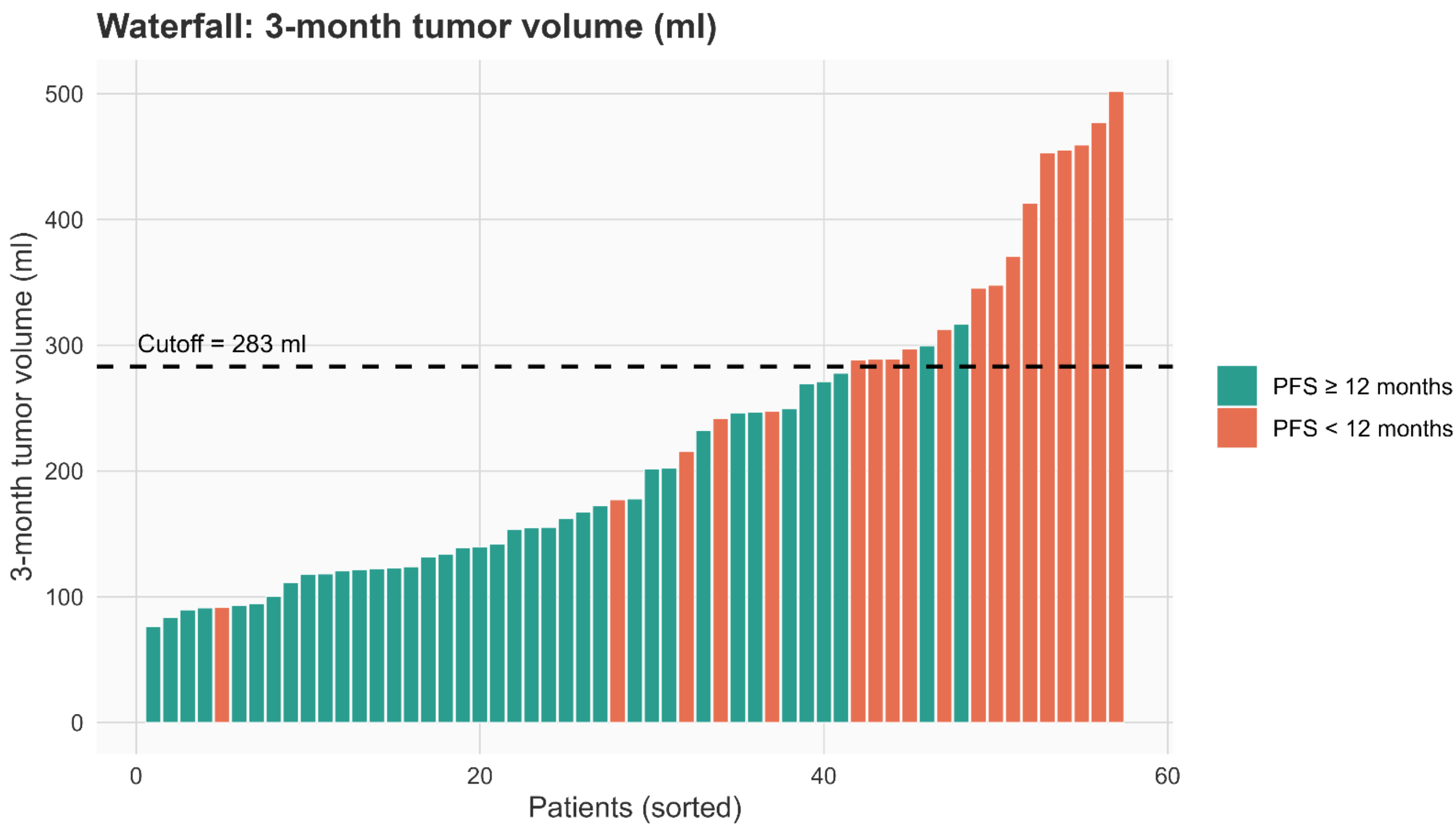

Figure S4. Waterfall plots of tumor volume by PFS group.
3-month tumor volumes sorted ascending; dashed line indicates cutoff 283 mL. Bars are colored by PFS group (PFS ≥ 12 months vs PFS < 12 months).
